# Fronto-striatal circuits for cognitive flexibility in far from onset Huntington’s disease: evidence from the Young Adult Study

**DOI:** 10.1136/jnnp-2020-324104

**Published:** 2020-10-31

**Authors:** Christelle Langley, Sarah Gregory, Katie Osborne-Crowley, Claire O'Callaghan, Paul Zeun, Jessica Lowe, Eileanoir B Johnson, Marina Papoutsi, Rachael I Scahill, Geraint Rees, Sarah J Tabrizi, Trevor W Robbins, Barbara Jacquelyn Sahakian

**Affiliations:** 1 Department of Psychiatry, University of Cambridge, Cambridge, UK; 2 Huntington’s Disease Centre, Department of Neurodegenerative disease, Institute of Neurology, University College London, London, UK; 3 Division of Equity, Diversity and Inclusion, University of New South Wales, Sydney, New South Wales, Australia; 4 Brain and Mind Centre, Faculty of Medicine and Health, The University of Sydney, Sydney, New South Wales, Australia; 5 University College London Institute of Cognitive Neuroscience, UCL, London, UK; 6 Department of Psychology and Behavioural and Clinical Neuroscience Institute, University of Cambridge, Cambridge, UK

## Abstract

**Objectives:**

Cognitive flexibility, which is key for adaptive decision-making, engages prefrontal cortex (PFC)-striatal circuitry and is impaired in both manifest and premanifest Huntington’s disease (pre-HD). The aim of this study was to examine cognitive flexibility in a far from onset pre-HD cohort to determine whether an early impairment exists and if so, whether fronto-striatal circuits were associated with this deficit.

**Methods:**

In the present study, we examined performance of 51 pre-HD participants (mean age=29.22 (SD=5.71) years) from the HD Young Adult Study cohort and 53 controls matched for age, sex and IQ, on the Cambridge Neuropsychological Test Automated Battery (CANTAB) Intra-Extra Dimensional Set-Shift (IED) task. This cohort is unique as it is the furthest from disease onset comprehensively studied to date (mean years=23.89 (SD=5.96) years). The IED task measures visual discrimination learning, cognitive flexibility and specifically attentional set-shifting. We used resting-state functional MRI to examine whether the functional connectivity between specific fronto-striatal circuits was dysfunctional in pre-HD, compared with controls, and whether these circuits were associated with performance on the critical extradimensional shift stage.

**Results:**

Our results demonstrated that the CANTAB IED task detects a mild early impairment in cognitive flexibility in a pre-HD group far from onset. Attentional set-shifting was significantly related to functional connectivity between the ventrolateral PFC and ventral striatum in healthy controls and to functional connectivity between the dorsolateral PFC and caudate in pre-HD participants.

**Conclusion:**

We postulate that this incipient impairment of cognitive flexibility may be associated with intrinsically abnormal functional connectivity of fronto-striatal circuitry in pre-HD.

## Introduction

Huntington’s disease (HD) is an inherited, rare, neurodegenerative disease characterised by movement, cognitive and psychiatric symptoms.^1^ HD is caused by a repeat expansion of the trinucleotide cytosine-adenine-guanine (CAG) in exon 1 of the Huntingtin gene (HTT) that leads to expression of a mutant form of the Huntingtin protein.^1^ The greater the number of CAG repeats, the earlier the HD onset.^2^ A diagnosis of HD is based on the presence of significant motor abnormalities. Premanifest HD (pre-HD) are gene carriers with increased CAG repeats, but without the presence of motor symptoms. Multiple studies have demonstrated that pre-HD participants already show cognitive, psychiatric and brain abnormalities, which can be detected up to 15 years before diagnosis.^3 4^ Models based on age and CAG repeats can assist in the prediction of the onset of motor symptoms,^2^ allowing the study of individuals decades before predicted onset.

It is well established that the neurodegeneration in both HD and pre-HD is especially severe in the striatum in HD,^3 5–8^ largely due to loss of GABAergic spiny projection neurons (medium spiny neurons).^9^ In more advanced stages of the disease, neurodegeneration becomes more widespread in the cortex.^5 10^ Indeed, fronto-striatal circuits are among the earliest to show degeneration in pre-HD.^10^ Moreover, studies of functional MRI (fMRI) in HD have reported abnormal patterns of activation in these fronto-striatal circuits, across a number of tasks.^11 12^ Studies of functional connectivity, which represents a measure of connectivity between brain regions, have shown a similar susceptibility of impairment in fronto-striatal circuits. Functional connectivity during both resting-state^13^ and task-related^14 15^ studies was abnormal in fronto-striatal circuits in pre-HD.

Performance on tests of cognitive flexibility is sensitive to disruption of fronto-striatal circuitry.^16–21^ Cognitive flexibility is vital for adaptive decision-making in everyday life. There have been only a few studies to examine the neural mechanisms of cognitive flexibility in HD. An early study demonstrated increased frontal blood flow in patients with HD during performance of the Wisconsin Card Sorting Test (WCST),^22^ which correlated positively with caudate atrophy.^11^ Both manifest and pre-HD patients showed increased activation in prefrontal and striatal regions, and this increased activation was associated with reduced errors in shifting responding in another test of cognitive flexibility.^23^ The Cambridge Neuropsychological Test Automated Battery (CANTAB) Intra-Extra Dimensional Set Shift (IED) test is another test of cognitive flexibility that has shown impairments in all phases of HD,^24–27^ including in premanifest patients.^26^ In fact, in early HD, the impairment in cognitive flexibility is even greater than in patients with frontal lobe damage of a similar age.^25^ To our knowledge, no previous studies have examined the underlying neural substrates of these deficits, although two studies have examined resting-state functional connectivity and performance on the CANTAB IED task, one in a healthy population^19^ and one in obsessive-compulsive disorder (OCD).^21^ In the healthy population, extradimensional (ED) shifting performance was correlated with resting-state functional connectivity between the dorsolateral prefrontal cortex (PFC) and ventral striatum.^19^ By contrast, in an OCD population, who also have impaired cognitive flexibility, resting-state functional connectivity between the ventrolateral PFC and caudate was associated with ED shifting.^21^

In the present study, we examined performance on the CANTAB IED in a group of pre-HD participants, far from disease onset from the HD Young Adult Study (HD-YAS) cohort.^28^ The large HD-YAS cohort is the furthest cohort from disease onset studied to date. Specifically, we used resting-state fMRI to examine the association between the functional connectivity in predefined fronto-striatal circuits^19 21^ and separate performance on the ED shift stage of the CANTAB IED, with three main hypotheses: (1) decreased intrinsic functional connectivity between fronto-ventral striatal circuits is associated with increased ED errors in the HC group; (2) in pre-HD, any mild deficit in ED errors is associated with reduced functional connectivity of the same circuit, or (3) alternatively in pre-HD, ED shifting is associated with an alternative fronto-striatal circuit, by analogy with what has been demonstrated for OCD.^21^

## Methods

### Participants

One hundred and thirty-one participants (64 pre-HD and 67 controls),^28^ closely matched for age, gender and IQ (measured by the National Adult Reading Test (NART)), were recruited from across the UK as part of the HD-YAS (online supplemental table 1). All participants were assessed at the National Hospital for Neurology and Neurosurgery, London, UK, by an experienced HD clinician. Pre-HD participants did not show clinical signs of HD: all had a Unified Huntington’s Disease Rating Scale Total Motor Score (UHDRS TMS)^29^ of ≤5, indicating a distinct lack of motor symptoms. Disease burden score,^30^ a product of age and HTT CAG repeats, was ≤240, which approximates to >18 years from predicted onset. CAG repeats were measured at a single laboratory for statistical analysis. Controls were either gene negative family members or individuals with no HD risk (partners or friends of HD gene carriers or members of the wider HD community). Inclusion and exclusion criteria are supplied in the online supplemental material. Additionally, 12 left-handed subjects were excluded from the present study. Therefore, a subset of 104 right-handed participants (51 pre-HD and 53 controls) who completed resting-state fMRI and were included in the present study, demographics are displayed in table 1.

10.1136/jnnp-2020-324104.supp1 Supplementary data

**Table 1:** Demographics

	Premanifest HD (n=51)	Healthy controls (n=53)	t value	P value
Age	29.22 (5.71)	28.85 (5.50)	−0.33	0.74
IQ (NART)	103.78 (8.17)	103.08 (7.37)	−0.47	0.64
Sex	51% Females (26)	58.5% Females (31)	0.76	0.45
CAG	42.10 (1.72), 39–47			
Years to onset^2^	23.89 (5.95), 10.02–36.12			

CAG, cytosine-adenine-guanine; HD, Huntington’s disease; NART, National Adult Reading Test.

### CANTAB intra-extra dimensional set shift task

**Figure 1:**
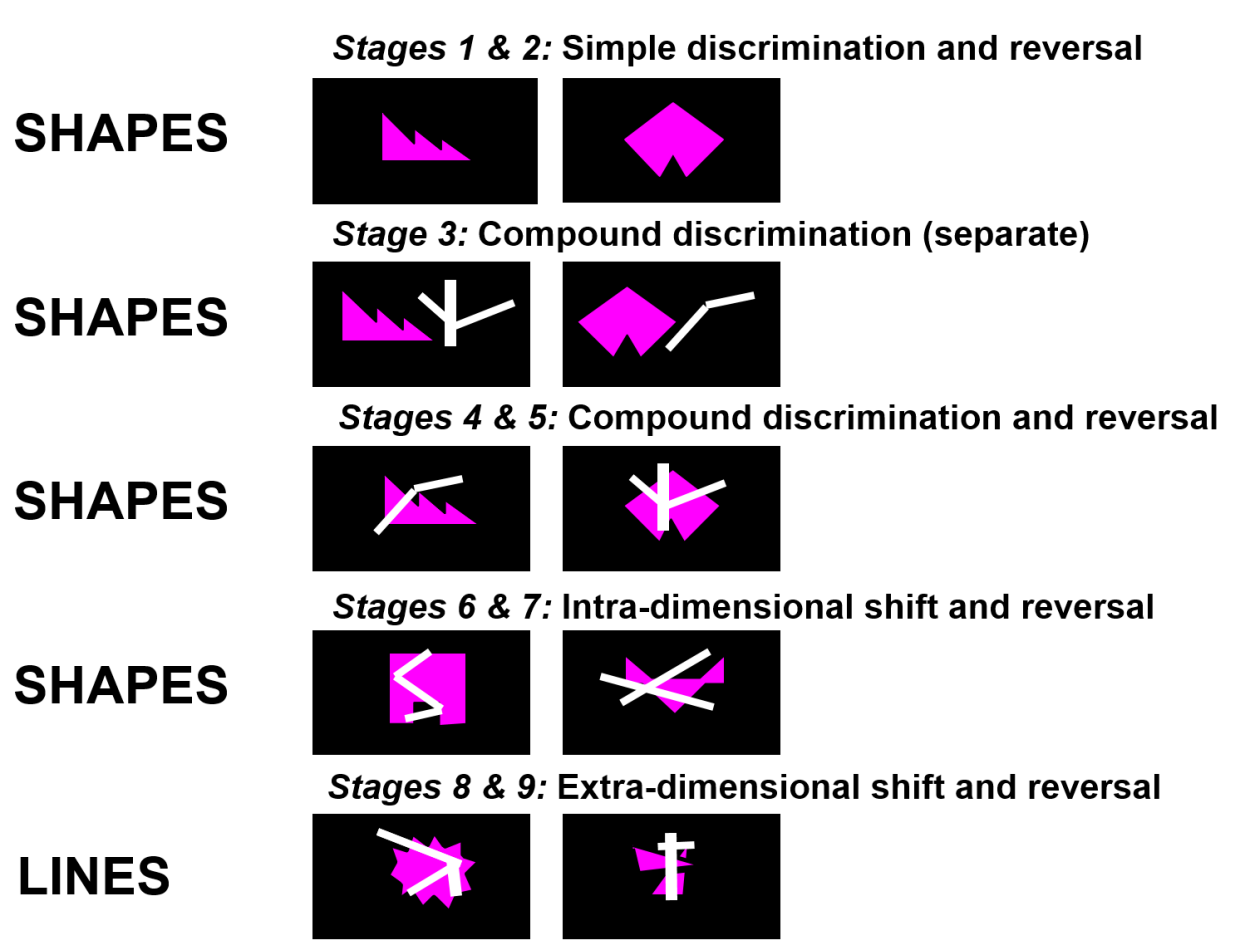
Schematic of the Cambridge Neuropsychological Test Automated Battery (CANTAB) Intra-Extra Dimensional Set-Shift (IED) Task.

A schematic of the CANTAB IED is presented in figure 1 and a full task description is provided in the online supplemental material.

### Behavioural analysis

Separate analyses of covariance (ANCOVAs) controlling for age, sex and IQ were conducted between pre-HD and HC groups to compare pre-ED errors and ED errors. In addition, we conducted a repeated measures ANCOVA with task (pre-ED or ED) as the within-group variable and group as the between-group variable. In the pre-HD group, only a partial correlation, controlling for age, IQ and sex, between CAG repeats and predicted years to onset and pre-ED and ED errors was conducted. To control for multiple comparisons, the Benjamini-Hochberg^31^ procedure was applied and the false discovery rate (FDR) set a priori at q<0.10. Original p values are reported and effect sizes are reported as partial eta squared (η$\frac{p}{2}$).

### Image acquisition

All MRI data were acquired on a 3T Prisma scanner (Siemens Healthcare, Germany) with radiofrequency body coil for transmission and a 64-channel head coil for signal reception using a protocol optimised for this cohort.^28^ The T1-weighted images were acquired using a 3D MPRAGE sequence with a repetition time (TR)=2530 ms and time to echo (TE)=3.34 ms; inversion time of 1100 ms, flip angle of 7°, field of view=256 mm^2^, 64 slices of 1.0 mm thickness were collected. The resting-state T2*-weighted images were acquired with a TR=3360 ms and TE=30 ms; field of view=192 mm^2^, flip angle of 90°, 48 slices of 2.5 mm thickness were collected anterior to posterior in the transverse orientation.

### Image preprocessing

All the functional images were preprocessed in SPM12 (http://www.fil.ion.ucl.ac.uk/spm). Images were slice-timing corrected, realigned, co-registered, normalised to Montreal Neurological Institute (MNI) space using the DARTEL deformation parameters from the segmentation of the T1 and Gaussian smoothing using 6 mm a full-width half-maximum Gaussian kernel. We specified eight regions of interest (ROIs) based on the coordinates identified in Morris *et al* 19 and Vaghi *et al* 21 (left and right caudate (±12, 6, 14), ventral striatum (±11, 12, –10), dorsolateral PFC (±33, 35, 36) and ventrolateral PFC (±20, 61, –4)). These regions were found to be associated with ED shifting in previous studies on the CANTAB IED in healthy individuals^19^ and patients with OCD.^21^ A 6 mm sphere was created at each coordinate. We used the FSL motion outliers function to determine the framewise displacement of each image. We determined that participants with mean FD >0.20 mm would be excluded.^32^ Movement was small in the cohort, and no participants were excluded. Following the preprocessing steps, noise from white matter, cerebrospinal fluid and movement signals were regressed out using least squares multiple regression, from each voxel. An additional linear detrending was applied to reduce spurious correlations. A bandpass filter (0.01–0.08) was applied to remove low-frequency and high-frequency noise. A mean time series was then extracted from each of the eight ROIs. The functional connectivity between ROIs was measured using Pearson’s correlation, resulting in an 8×8 weighted connectivity matrix for each participant. To increase the normality and standardise the data for group comparison, a Fisher z-transform was conducted. These values from the standardised weighted connectivity matrices were used to perform the correlation analyses with ED errors in IBM SPSS Statistics for Windows V.26 (IBM Corp, Armonk, NY, USA.).

### Network analysis

A multivariate analysis of covariance, controlling for age, sex and IQ, was conducted to compare the pairwise functional connectivity between the pre-HD and the HC groups. Partial correlations, controlling for age, sex and IQ, were conducted between ED errors, as well as CAG repeats, and the pairwise ROI functional connectivity between the striatal and frontal regions (ie, left caudate—left ventrolateral PFC; left caudate—right ventrolateral PFC; left caudate—left dorsolateral PFC; and left caudate—right dorsolateral PFC). Differences between the pre-HD and control group in correlation coefficients were compared using the *cocor* package in R. The Benjamini-Hochberg^31^ procedure was applied for each group (pre-HD and HC) and the FDR set a priori at q<0.10. Original p values are reported.

## Results

### Behavioural results

#### Group comparison

**Figure 2:**
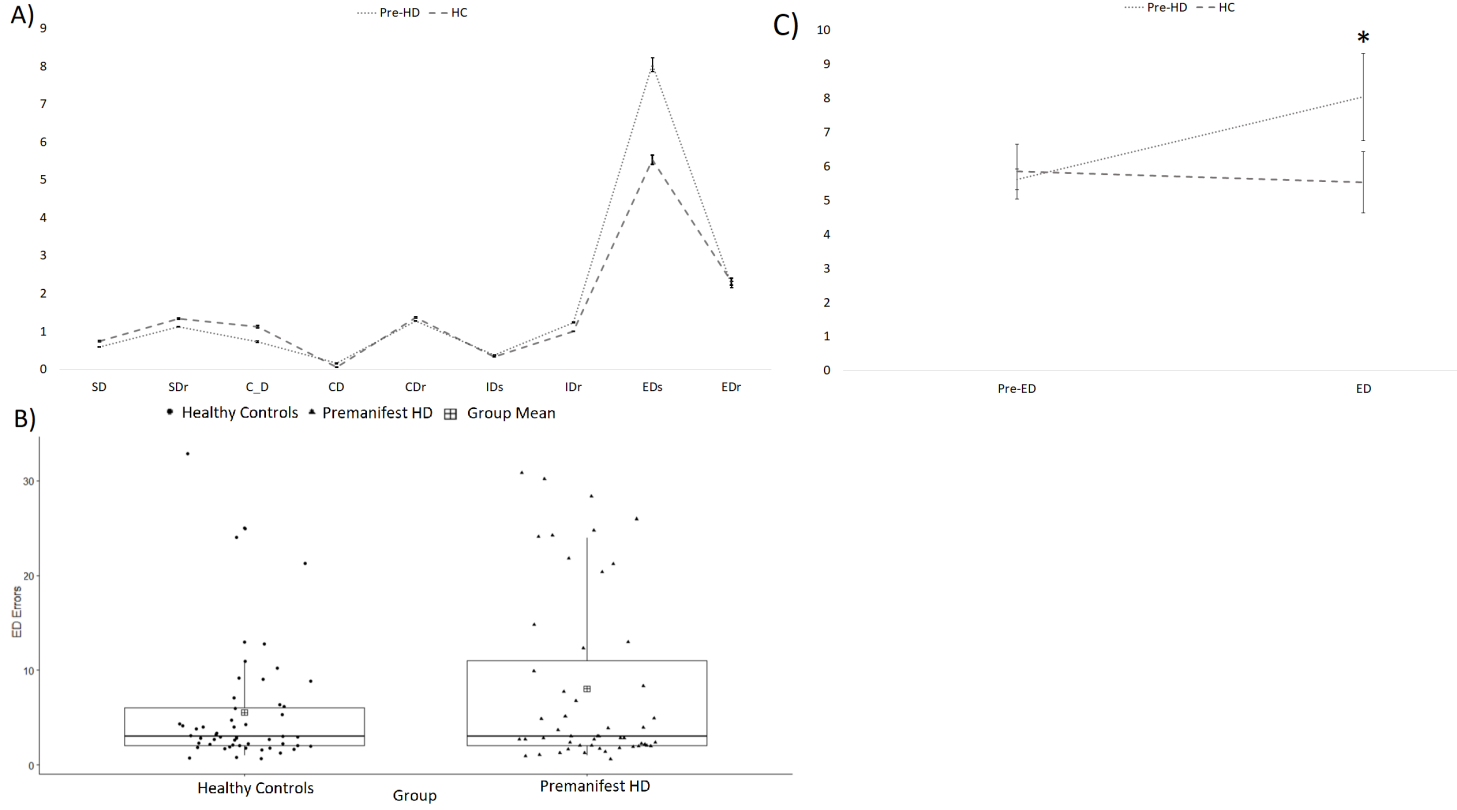
Performance on the Cambridge Neuropsychological Test Automated Battery (CANTAB) Intra-Extra Dimensional Set-Shift (IED) Task. (A) The mean number of errors by learning stage on the IED task. Error bars represent standard error of the mean. *p < 0.05. CD, superimposed compound discrimination; C_D, separated compound discrimination; CDr, superimposed compound discrimination reversal; EDs, extradimensional shift; EDr, extradimensional shift reversal; IDs, intradimensional shift; IDr, intradimensional shift reversal; SD, simple discrimination; SDr, simple discrimination reversal. (B) A boxplot of the extradimensional shift errors specifically. (C) A line graph displaying the mean pre-ED and ED errors in each group. Error bars represent standard error of the mean. ED, extradimensional; HC, healthy controls; HD, Huntington’s disease.

The ANCOVA showed that the pre-HD group made significantly more ED shift errors than the HC (F(1,99)=4.33, p=0.04, η$\frac{p}{2}$ =0.04) but there were no significant differences between the groups for pre-ED errors (F(1,99)=.40, p=0.53, η$\frac{p}{2}$ <0.01). The repeated measures ANCOVA showed a trend toward significance for both the main effect of group (F(1,99)=3.52, p=0.06, η$\frac{p}{2}$ =0.03) and the main effect of task (F(1,99)=3.85, p=0.053, η$\frac{p}{2}$ =0.04). There was a significant interaction effect of Task × Group (F(1,99)=4.46, p=0.04, η$ŋ\frac{p}{2}$ =0.04), where the pre-HD group made more ED errors than HC, but not more pre-ED errors (figure 2).

Within the pre-HD group, there were no significant correlations between CAG repeats and pre-ED errors (R=−0.22, p=0.13) or ED errors (R=−0.02, p=0.87). Similarly, there were no significant correlations between predicted years to onset and pre-ED errors (R=0.22, p=0.14) or ED errors (R=0.06, p=0.70).

### Functional connectivity results

#### Group comparison

There were no significant differences in functional connectivity for any of the pairwise ROIs between pre-HD and the HC group (see online supplemental table 2).

#### Correlation with ED errors

**Figure 3:**
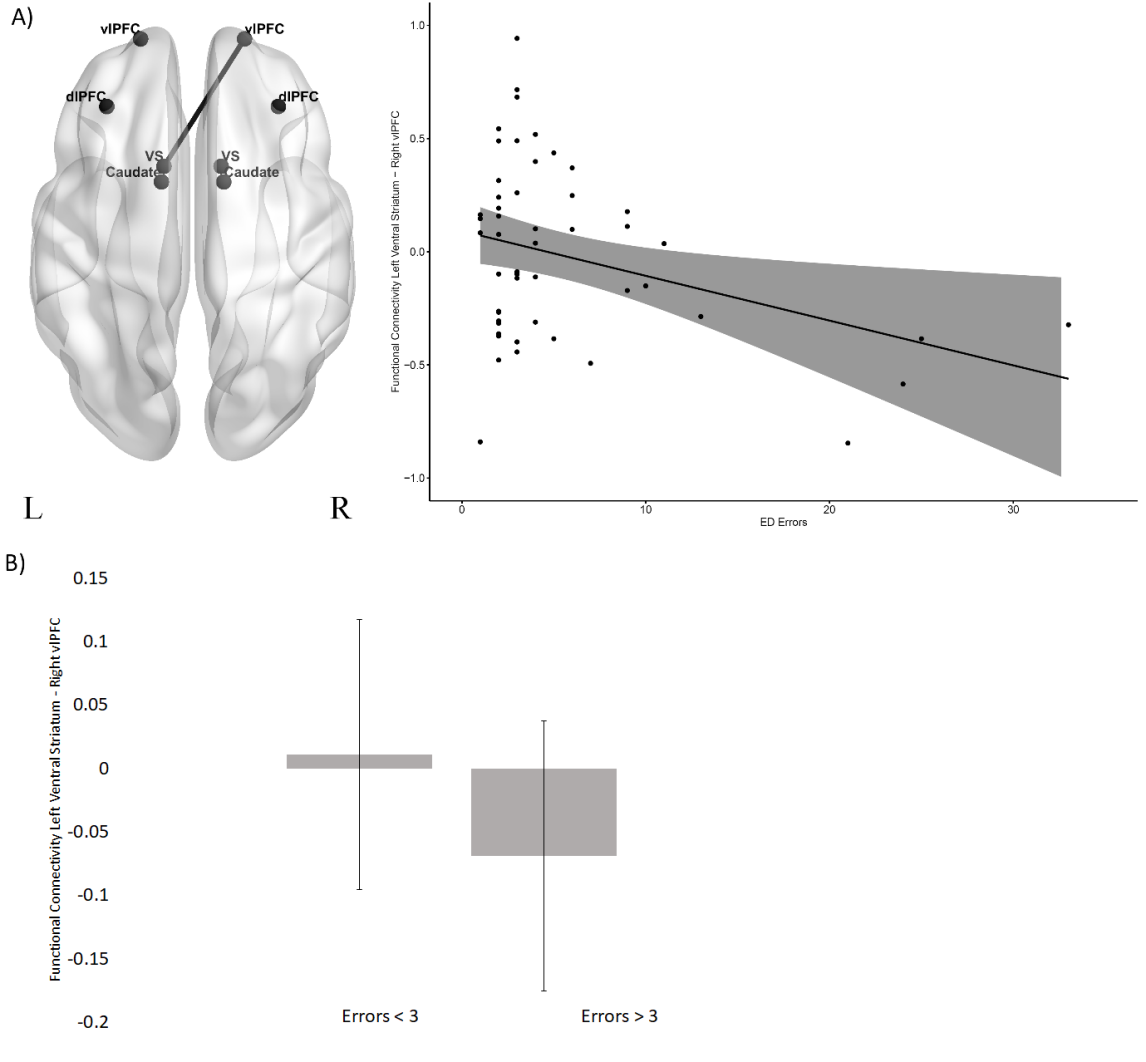
Scatterplot between functional connectivity and ED errors in the HC group. (A) The scatterplot between ED errors and functional connectivity between the left ventral striatum and the right ventrolateral PFC. (B) A bar plot showing mean functional connectivity between the left ventral striatum and the right ventrolateral PFC in the HC group (median split according to ED errors). Error bars represent the standard error of the difference. dlPFC, dorsolateral PFC; ED, extradimensional; HC, healthy controls; PFC, prefrontal cortex; vlPFC, ventrolateral PFC.

**Figure 4:**
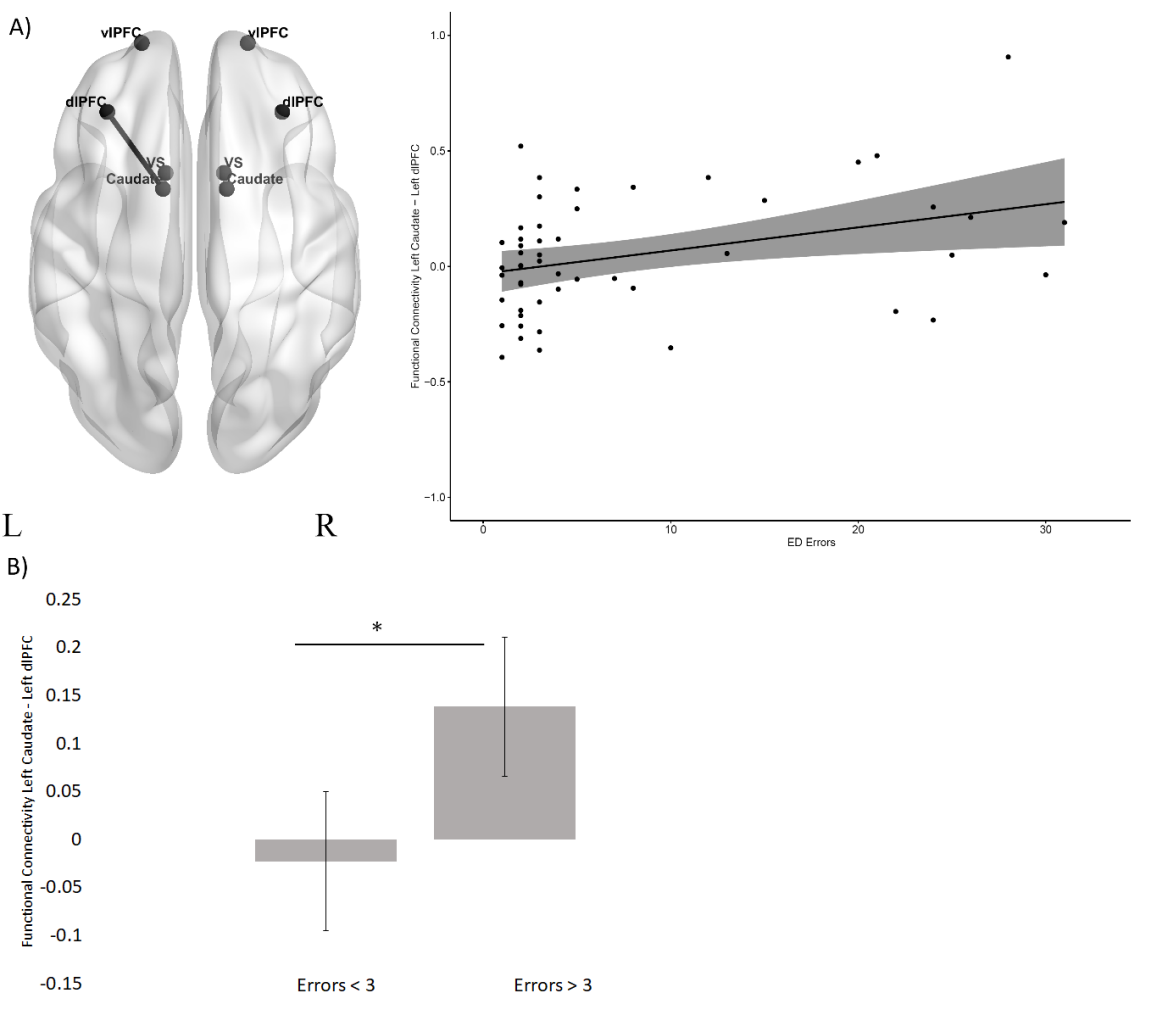
Functional connectivity and ED errors in premanifest HD group. (A) The scatterplot between ED errors and functional connectivity between the left caudate and left dorsolateral PFC. (B) A bar plot showing mean functional connectivity between the left caudate and the left dorsolateral PFC in patients with pre-HD (median split according to ED errors). Error bars represent the standard error of the difference. *p<0.05. dlPFC, dorsolateral PFC; ED, extradimensional; HD, Huntington’s disease; PFC, prefrontal cortex; vlPFC, ventrolateral PFC.

In the HC group (figure 3), there was a significant negative correlation between ED errors and functional connectivity between the left ventral striatum and the right ventrolateral PFC (R=−0.35, p=0.012). By contrast, for the pre-HD group (figure 4), there was a positive correlation between ED errors and functional connectivity between the left caudate and the left dorsolateral PFC (left R=0.40, p=0.004). In these two circuits, the correlation coefficients were significantly different between the HC and the pre-HD group (left ventral striatum and right ventrolateral PFC M_HC_=0.35, M_HD_=0.06, z=−2.11, p=0.017; left caudate and left dorsolateral PFC M_HC_=0.09, M_HD_=0.40, z=−1.68, p=0.05). As in Vaghi *et al*,^21^ we conducted a post hoc comparison of intrinsic resting-state functional connectivity in the HC and pre-HD group separately, between those who made above or below the median errors (3 ED errors). The results showed that in the HC group (figure 3) there was no significant difference in functional connectivity between left ventral striatum and right ventrolateral PFC between the two error groups (F(1,48)=.53, p=0.46, η$\frac{p}{2}$ =0.01). However, in the pre-HD group (figure 4), functional connectivity between left caudate and the left dorsolateral PFC was significantly higher in the above median errors group (F(1,46)=5.71, p=0.02, η$\frac{p}{2}$ =0.11). All results are presented in online supplemental table 3.

### Correlation with CAG repeats

There was no significant correlation between CAG repeats and any of the pairwise ROIs in the pre-HD group (online supplemental table 4).

## Discussion

We examined cognitive flexibility in a large group of far from onset pre-HD gene carriers from the HD-YAS.^28^ We hypothesised that there may be a small impairment at the ED shifting stage in this early pre-HD group. We predicted that decreased intrinsic functional connectivity between fronto-striatal circuits would be associated with increased ED errors in the HC group (hypothesis 1). In the pre-HD group, we expected either a similar negative association in the same circuit as the HC (hypothesis 2) or that ED shifting would be associated with an alternative fronto-striatal circuit (hypothesis 3). Indeed, the pre-HD group exhibited mild cognitive inflexibility compared with controls. The results from the functional connectivity analysis support the first of these hypotheses in showing a negative correlation between ED errors and intrinsic functional connectivity between the ventrolateral PFC and ventral striatum in controls. By contrast, consistent with our third a priori hypothesis, the pre-HD group showed a positive association with errors in an alternative fronto-striatal circuit, between the dorsolateral PFC and caudate.

Our behavioural results demonstrated that the pre-HD group successfully formed attentional sets and achieved reversal learning but had a specific impairment in shifting attentional control between stimulus dimensions when compared with controls. Previous studies have shown similar impairment in set-shifting in HD. Patients with HD made more perseverative errors on the WCST.^24^ The impairment was specific to shifting and not in forming or maintaining a set. Similarly, a deficit in ED shifting on the CANTAB IED task in patients with early manifest HD has been reported.^25^ In fact, in that study fewer than 20% of participants were able to reach the learning criterion of six consecutive correct responses in the 50 trials. A pre-HD group showed a more modest, but still statistically significant impairment in ED shifting.^26^ Although we also found a significant difference between the pre-HD and control groups in the present study, a large proportion of the pre-HD group performed the task as well as controls with only a small number of pre-HD participants performing poorly. This result is supported by the relatively low mean number of ED errors in the current sample (8.04) of pre-HD participants compared with previous data for a pre-HD group closer to disease onset (~15 mean errors^26^). Tests of cognitive flexibility such as the CANTAB IED and the WCST may be among those most sensitive for detecting impairments in pre-HD participants. Indeed, early converters, receiving a clinical diagnosis, perform worse on the WCST compared with both late and non-converters.^33^ In the study by Brandt *et al.* 33 patients with pre-HD were closer to conversion than in our study, which to our knowledge, is the earliest pre-HD group used to date by approximately 5 years. The results highlight the sensitivity of the CANTAB IED task, specifically the ED shift stage, for early detection of cognitive impairment in HD. However, the present mild, but somewhat specific, impairment in cognitive flexibility in far from onset HD patients has to be considered in the wider context of an absence of overall cognitive deficits in this same pre-HD group when subjected to a more extensive test battery examining other cognitive and emotional domains.^28^

In support of our hypothesis, the functional connectivity analysis showed a significant negative correlation between ED errors and intrinsic functional connectivity between the left ventral striatum and the right ventrolateral PFC in the HC group. Studies have implicated the ventral striatum in set-shifting in both humans^19 20^ and rodents.^16^ Involvement of the ventrolateral PFC in attentional set-shifting has also been demonstrated in neuroimaging studies of healthy control volunteers and patients with OCD.^34^ A meta-analysis showed lateral PFC involvement during WCST and specifically ventrolateral PFC during task-switching.^17^ This is further supported by the animal literature, where excitotoxic lesions of the lateral PFC in marmosets impaired attentional set-shifting.^18^ The results from the present study provide further evidence for the involvement of fronto-striatal networks in cognitive flexibility, specifically between the PFC and the ventral striatum, in healthy individuals. The lateralisation of this effect has not been explored further in this study as this did not form part of our initial hypotheses. In experimental studies, rats with unilateral lesions are able to compensate behaviourally, likely by employing the contralateral hemisphere, whereas crossed prefrontal and striatal lesions result in a complete behavioural deficit, similar to that of bilateral lesions.^35^ However, future studies designed to rigorously and statistically test the lateralisation effect will be required to elucidate whether attentional set-shifting may depend on interhemispheric communication.

In the pre-HD group, an alternative fronto-striatal network was associated with ED shifting performance. There was a positive correlation between ED errors and the functional connectivity between the left caudate and the left dorsolateral PFC. Patients with OCD, who also have impaired cognitive flexibility, similarly showed that an alternative fronto-striatal connectivity was related to the ED shift stage of the CANTAB IED^21^ although the circuit implicated connected the caudate with the ventrolateral PFC. Previous studies on experimental animals have shown that caudate lesions have only limited effects on ED shifting.^36^ Similarly, in healthy human participants, caudate activation was associated with rule reversal rather than ED shifting.^37^ Moreover, the study of Morris *et al* 19 implicated the ventral striatum rather than the caudate in ED shifting. Hence, an involvement of the caudate nucleus is not typically present in performing the ED shift.

However, there is an association of the caudate nucleus with ED shift performance in both HD and OCD, which may result from some form of functional reorganisation of fronto-striatal circuitry. In the present case, resting-state functional connectivity between the caudate and the dorsolateral PFC is associated with performance of the ED shift in pre-HD participants, outside of the scanner, unlike in controls. Previous studies have suggested that increased task-based activity^23 38 39^ and increased resting-state functional coupling^39^ may represent some form of compensatory activity that maintains task performance in HD. Indeed, Gray *et al* 23 found evidence for early functional compensation in fronto-striatal circuits in pre-HD. Thus, the functional reorganisation of fronto-striatal circuitry in pre-HD observed in the present study could have possible functional compensatory effects; this is supported by the fact that many of the pre-HD group had normal levels of performance linked to functional connectivity in this dorsolateral PFC-caudate circuit. However, confirmation of this hypothesis would require formal testing. Including the relatively small number of pre-HD individuals with significantly impaired ED shifting, there was a striking positive relationship with functional connectivity of this circuitry, suggesting possible maladaptive, rather than compensatory, influences. Why such functional reorganisation would necessarily be associated with impairments in cognitive flexibility is a matter for speculation. There is some evidence that different fronto-striatal circuits may sometimes compete in control of behavioural output.^40^ It is possible that the involvement of the dorsolateral PFC with the caudate in the pre-HD group represents an inefficient strategy based on increased searching for overelaborate, and hence counterproductive, rules or solutions governing performance in the IED task rather than responding appropriately to reinforcing feedback.^34^

In the present study, we correlated resting-state functional connectivity with behavioural performance measured outside the scanner in order to provide direct correlations of brain states with cognitive performance, which presumably represent the pre-existing capability of the fronto-striatal circuitry to mediate ED shifting, and hence cognitive flexibility. However, our results also showed considerable overlap with those regions observed in task-based fMRI studies on the CANTAB IED^34^ as well as in animal studies, which can more readily test causal relationships.^18^ Therefore, task performance is clearly dependent to some extent on intrinsic functional connectivity at rest, reflecting the important influence of prior neural states. Indeed, our results suggest that impaired cognitive flexibility in a pre-HD group, far from onset, is associated with altered intrinsic functional connectivity between the caudate and dorsolateral PFC. While these findings were unrelated to CAG repeats, they provide the potential for a neuroimaging biomarker of individual variability in cognitive flexibility in pre-HD, even at an early stage in disease progression.

We suggest two potential future directions to better elucidate the function of fronto-striatal networks in cognitive flexibility. As HD disease progression continues, the impairment appears to shift from one form of cognitive flexibility to another, from deficits in attentional set-shifting to reversal learning, which impacts especially on responding to reinforcing feedback.^27^ As such, future studies examining the neural substrates that underlie this later reversal impairment could allow for further understanding of how fronto-striatal networks are differentially impacted during the course of a progressive neurodegenerative disorder. In addition, examining both deterministic and probabilistic reversal learning paradigms and their neural substrates could further differentiate the function of these fronto-striatal circuits.

## Conclusion

The present study demonstrated that the CANTAB IED task detects a mild impairment in cognitive flexibility in a pre-HD group far from disease onset. The majority of the pre-HD sample performed comparably with controls, but a small number of participants performed less well. In healthy individuals, functional connectivity between the ventrolateral PFC and ventral striatum is associated with cognitive flexibility. In the pre-HD group, alternative fronto-striatal circuits were associated with attentional set-shifting, potentially representing a form of functional reorganisation, which while effective for most pre-HD participants in preserving performance is maladaptive in a small number of the most affected pre-HD participants. The intrinsic functional connectivity at rest in relation to performance on this test of cognitive flexibility may thus provide a potential neuroimaging biomarker of individual variability in cognitive flexibility in pre-HD early in disease progression.
