# Supplementary material for: Fronto-striatal circuits for cognitive flexibility in far from onset Huntington’s disease: evidence from the Young Adult Study
Source: J Neurol Neurosurg Psychiatry. 2020 Oct 31;92(2):143–9. doi: 10.1136/jnnp-2020-324104 (PMC7841479; doi:10.1136/jnnp-2020-324104)
Supplement: Supplementary data [file jnnp-2020-324104supp001.pdf]

Supplemental Methods

Participants

Supplementary Table 1 displays the demographic data for the full sample reported in Scahill et al. (2020) [1].

Supplementary Table 1. Demographics.

	Premanifest HD (N = 64)	Healthy Controls (N = 67)	P value
Age	29.0 (5.60)	29.10 (5.70)	.95
Sex	53% Females (34)	57% Females (38)	.81
IQ	102.40 (7.50)	103.50 (8.30)	.42
Years to onset	23.60 (5.80)	N/A	

Participants were identified and recruited from the Enroll-HD study https://www.enroll-hd.org/, regional Genetic and HD centres across the UK who were established as patient identification sites, and through broader efforts such as via the Huntington's Disease Association https://www.hda.org.uk/ and the Huntington's Disease Youth Organisation https://hdyo.org/.

Eligibility screening

Prior to enrolment, each participant was interviewed to determine whether they meet the eligibility criteria below. For gene carriers, CAG expansion in the HD gene was confirmed by obtaining the genetic report from an accredited laboratory. This was to confirm CAG expansion and standardise CAG sizing for statistical analysis.

Inclusion criteria

a. Are 18-40 years of age, inclusive; and

b. Are capable of providing informed consent and

c. Are capable of complying with study procedures and

For the **Healthy Control** group, participants eligible are persons who meet the following criteria:

Langley C, *et al. J Neurol Neurosurg Psychiatry* 2021; 92:143–149. doi: 10.1136/jnnp-2020-324104

d. Have no known family history of HD (family control or community control); or

e. Have known family history of HD but have been tested for the huntingtin gene CAG expansion and are not at genetic risk for HD (CAG < 36*) (gene negative).

For the **Young Adult Premanifest HD** group, participants eligible are persons who meet the additional following criteria:

f. Do not have clinical diagnostic motor features of HD, defined as Unified Huntington's Disease Rating Scale (UHDRS) Diagnostic Confidence Score [2] < 4; and

g. Have CAG expansion ≥ 40*; and

h. A disease burden score (DBS) [3] ≤ 240**[2]

The rationale for this DBS cut-off is that this boundary corresponds approximately to >18 years to estimated disease onset according to the Langbehn formula [4].

Exclusion criteria

a. Current use of investigational drugs or participation in a clinical drug trial within 30 days prior to study visit; or

b. Current intoxication, drug or alcohol abuse or dependence; or

c. If using any antidepressant, psychoactive, psychotropic or other medications or nutraceuticals used to treat HD, the use of inappropriate (e.g., non-therapeutically high) or unstable dose within 30 days prior to study visit; or

d. Significant medical, neurological or psychiatric co-morbidity likely, in the judgment of the Principal Investigator, to impair participant’s ability to complete essential study procedures; or

e. Predictable non-compliance as assessed by the Principal Investigator; or

f. Inability or unwillingness to undertake any of the essential study procedures; or

g. Needle phobia; or

h. Contraindication to MRI, including, but not limited to, MR-incompatible pacemakers, recent metallic implants, foreign body in the eye or other indications, as assessed by a standard pre-MRI questionnaire; or

i. Pregnant (as confirmed by urine pregnancy test); or

j. Claustrophobia, or any other condition that would make the subject incapable of undergoing an MRI.

For the optional CSF collection only

k. Needle phobia, frequent headache, significant lower spinal deformity or major surgery; or

l. Antiplatelet or anticoagulant therapy within the 14 days prior to sampling visit, including but not limited to: aspirin, clopidogrel, dipyridamole, warfarin, dabigatran, rivaroxaban and apixaban; or

m. Clotting or bruising disorder; or

n. Screening blood test results outside the clinical laboratory's normal range for the following: white cell count, neutrophil count, lymphocyte count, haemoglobin (Hb), platelets, prothrombin time (PT) or activated partial thromboplastin time (APTT); or

o. Screening blood test results for C-reactive protein (CRP)>2× upper limit of normal; or

p. Exclusion during history or physical examination, final decision to be made by the Principal Investigator; including but not limited to:

i any reason to suspect abnormal bleeding tendency, e.g. easy bruising, petechial rash; or

ii any reason to suspect new focal neurological lesion, e.g. new headache, optic disc swelling, asymmetric focal long tract signs; or

iii any other reason that, in the clinical judgment of the operator or the Principal Investigator, it is felt that lumbar puncture is unsafe.

CANTAB Intra-Extra Dimensional Set Shift Task

The CANTAB IED[5] is a 7-minute test measuring cognitive flexibility and has similarities to a computerised version of the Wisconsin Card Sorting Test[6]. The task initially features rule acquisition and reversal learning and then attentional set formation and set shifting. Participants are presented with two artificial dimensions including pink shapes and white lines.

Through trial and error the participant must select the correct rule. After six correct responses, the stimuli and/or rule changes. There are nine stages to the test. Initially the test presents simple stimuli with just one dimension (pink shapes). These later change to compound stimuli (white lines overlaid on pink shapes). Early in the test the shifts are intra-dimensional (ID) (pink shapes are relevant) to establish set formation. This stage assesses generalisation of learning. Then at stage 8 a crucial extra-dimensional (ED) shift occurs, white lines become relevant (attentional set-shifting). This stage assesses cognitive flexibility. This latter stage is followed by a final reversal of the rule. Importantly, the task is not time-limited and participants have 50 trials to reach learning criterion. Outcomes measures for the present study include the number of pre-ED errors (stages 1-7) and the ED shift errors (stage 8). Pre-ED errors measure learning performance on the stages up to but not including the critical ED shifting stage.

Supplemental Results

Separate analysis of covariance (ANCOVAs), controlling for age, sex and IQ were conducted between pre-HD and HC groups to compare pre-ED latency and ED latency. The results showed that there was no statistically significant difference between pre-HD and HC groups on either the pre-ED latency ($F(1,99)=.691$, $p=.408$ $\eta_p^2=.007$) or ED latency ($F(1,99)=.463$, $p=.498$ $\eta_p^2=.005$).

Full results for the functional connectivity comparison between pre-HD and HC groups are displayed in Supplementary Table 2.

Supplementary Table 2. Functional connectivity group comparisons.

	Premanifest HD	Healthy Controls	t-value	p-value	η_p^2
Right Caudate – Right vlPFC	.165 (.342)	.072 (.339)	1.836	.179	.018
Right Caudate - Left vlPFC	.084 (.352)	.081 (.307)	.012	.912	.001
Right Caudate – Right dlPFC	.008 (.297)	.024 (.300)	.073	.788	.001
Right Caudate - Left dlPFC	.015 (.279)	.031 (.300)	.029	.864	.001
Left Caudate – Right vlPFC	.135 (.333)	.060 (.337)	1.358	.247	.014
Left Caudate- Left vlPFC	.075 (.325)	.054 (.302)	.140	.709	.001
Left Caudate - Right dlPFC	.028 (.273)	-.023 (.351)	.631	.429	.006
Left Caudate - Left dlPFC	.049 (.261)	.024 (.316)	.196	.659	.002
Right Ventral Striatum – Right vlPFC	.118 (.345)	.016 (.368)	1.693	.196	.017
Right Ventral Striatum - Left vlPFC	.096 (.363)	.074 (.357)	.040	.841	.001
Right Ventral Striatum - Right dlPFC	-.107 (.286)	-.093 (.353)	.077	.782	.001
Right Ventral Striatum - Left dlPFC	-.089 (.260)	-.105 (.261)	.022	.883	.001
Left Ventral Striatum - Right vlPFC	.088 (.334)	-.018 (.390)	1.663	.200	.017
Left Ventral Striatum - Left vlPFC	.127 (.359)	.064 (.400)	.460	.499	.005
Left Ventral Striatum - Right dlPFC	-.115 (.274)	-.119 (.355)	.006	.938	.001
Left Ventral Striatum - Left dlPFC	-.079 (.259)	-.093 (.279)	.030	.864	.001

Langley C, *et al. J Neurol Neurosurg Psychiatry* 2021; 92:143–149. doi: 10.1136/jnnp-2020-324104

The full results for the partial correlations, controlling for age, IQ and sex, between ED errors and pairwise functional connectivity for both the pre-HD and HC groups are displayed in Supplementary Table 3.

Supplementary Table 3. Correlations between all pairwise ROIs and ED Errors.

	Premanifest HD		Healthy Control	
	ED Errors		ED Errors	
	R-Value	P-Value	R-Value	P-Value
Right Caudate – Right vlPFC	.066	.657	.351	.011
Right Caudate - Left vlPFC	-.243	.096	.091	.530
Right Caudate – Right dlPFC	.195	.185	.102	.479
Right Caudate - Left dlPFC	.329	.022	-.032	.826
Left Caudate – Right vlPFC	-.086	.559	.223	.120
Left Caudate- Left vlPFC	-.244	.095	.035	.808
Left Caudate - Right dlPFC	.140	.342	.130	.370
Left Caudate - Left dlPFC	.404	.004*	.089	.539
Right Ventral Striatum – Right vlPFC	.069	.642	-.306	.031
Right Ventral Striatum - Left vlPFC	.100	.500	0.009	.950
Right Ventral Striatum - Right dlPFC	-.100	.944	-.141	.330
Right Ventral Striatum - Left dlPFC	-.190	.195	-.141	.330
Left Ventral Striatum - Right vlPFC	.064	.666	-.348	.012*
Left Ventral Striatum - Left vlPFC	.009	.950	-.111	.444
Left Ventral Striatum - Right dlPFC	-.042	.775	-.049	.737
Left Ventral Striatum- Left dlPFC	-.221	.130	-.055	.706

Note: * Survived FDR correction

Langley C, *et al. J Neurol Neurosurg Psychiatry* 2021; 92:143–149. doi: 10.1136/jnnp-2020-324104

The full results for the partial correlations, controlling for age, IQ and sex, between CAG repeats and pairwise functional connectivity for both the pre-HD group are displayed in Supplementary Table 4.

Supplementary Table 4. Correlations between all pairwise ROIs and CAG repeats in the pre-HD group.

	Premanifest HD	
	R-Value	P-Value
Right Caudate – Right vlPFC	-.014	.925
Right Caudate - Left vlPFC	-.039	.794
Right Caudate – Right dlPFC	.076	.607
Right Caudate - Left dlPFC	.167	.257
Left Caudate – Right vlPFC	-.032	.828
Left Caudate- Left vlPFC	.013	.930
Left Caudate - Right dlPFC	.104	.483
Left Caudate - Left dlPFC	.166	.260
Right Ventral Striatum – Right vlPFC	-.033	.823
Right Ventral Striatum - Left vlPFC	.001	.996
Right Ventral Striatum - Right dlPFC	.161	.276
Right Ventral Striatum - Left dlPFC	.031	.833
Left Ventral Striatum - Right vlPFC	.049	.743
Left Ventral Striatum - Left vlPFC	.041	.780
Left Ventral Striatum - Right dlPFC	-.003	.986
Left Ventral Striatum- Left dlPFC	-.023	.879

Note: * Survived FDR correction

References

1. Scahill, R., Zeun, P., Osborne-Crowley, K., et al. Biological and clinical characteristics of gene carriers far from predicted onset in the Huntington's disease Young Adult Study (HD-YAS): a cross-sectional analysis. *Lancet Neurol* 2020;19:502-512.
2. Kieburtz, K., Penney, J.B., Corno, P., et al. Unified Huntington's disease rating scale: reliability and consistency. *Neurology* 2001;11:136-142.
3. Penney Jr, J.B., Vonsattel, J.P., Macdonald, M.E., et al. CAG repeat number governs the development rate of pathology in Huntington's disease. *Ann Neurol* 1997;41:689-692.
4. Langbehn, D.R., Hayden, M.R., Paulsen, J.S. et al. CAG-repeat length and the age of onset in Huntington disease (HD): a review and validation study of statistical approaches. *American Journal of Medical Genetics Part B: Neuropsychiatric Genetics* 2010;153:397-408.
5. CANTAB® [Cognitive assessment software]. Cambridge Cognition (2019).
6. Berg, E.A. A simple objective technique for measuring flexibility in thinking. *J Gen Psychol* 1948;39:15-22.
